# Cortical and subcortical morphological alteration in Angelman syndrome

**DOI:** 10.1186/s11689-022-09469-3

**Published:** 2023-02-14

**Authors:** Xiaonan Du, Lei Wei, Baofeng Yang, Shasha Long, Ji Wang, Aiqi Sun, Yonghui Jiang, Zhongwei Qiao, He Wang, Yi Wang

**Affiliations:** 1 Department of Neurology, Children’s Hospital of Fudan University, National Children’s Medical Center, Shanghai, China; 2 Institute of Science and Technology for Brain-inspired Intelligence, Fudan University, Shanghai, China; 3 Human Phenome Institute, Fudan University, Shanghai, China; 4 Department of Genetics and Paediatrics, Yale School of Medicine, CT New Haven, China; 5 Department of Radiology, Children’s Hospital of Fudan University, National Children’s Medical Center, Shanghai, China; 6 Key Laboratory of Computational Neuroscience and BrainInspired Intelligence (Fudan University), Ministry of Education, Shanghai, USA

**Keywords:** Angelman syndrome, MRI, Seizure, Brain morphometry

## Abstract

**Background:**

Angelman syndrome (AS) is a neurodevelopmental disorder with serious seizures. We aim to explore the brain morphometry of patients with AS and figure out whether the seizure is associated with brain development.

**Methods:**

Seventy-three patients and 26 healthy controls (HC) underwent high-resolution structural brain MRI. Group differences between the HC group and the AS group and also between AS patients with seizure (AS-Se) and age-matched AS patients with non-seizure (AS-NSe) were compared. The voxel-based and surface-based morphometry analyses were used in our study. Gray matter volume, cortical thickness (CTH), and local gyrification index (LGI) were assessed to analyze the cortical and subcortical structure alteration in the AS brain.

**Results:**

Firstly, compared with the HC group, children with AS were found to have a significant decrease in gray matter volume in the subcortical nucleus, cortical, and cerebellum. However, the gray matter volume of AS patients in the inferior precuneus was significantly increased. Secondly, patients with AS had significantly increased LGI in the whole brain as compared with HC. Thirdly, the comparison of AS-Se and the AS-NSe groups revealed a significant decrease in caudate volume in the AS-Se group. Lastly, we further selected the caudate and the precuneus as ROIs for volumetric analysis, the AS group showed significantly increased LGI in the precuneus and reduced CTH in the right precuneus. Between the AS-Se and the AS-NSe groups, the AS-Se group exhibited significantly lower density in the caudate, while only the CTH in the left precuneus showed a significant difference.

**Conclusions:**

These results revealed cortical and subcortical morphological alterations in patients with AS, including globally the decreased brain volume in the subcortical nucleus, the increased gray matter volume of precuneus, and the whole-brain increase of LGI and reduction of CTH. The abnormal brain pattern was more serious in patients with seizures, suggesting that the occurrence of seizures may be related to abnormal brain changes.

**Supplementary Information:**

The online version contains supplementary material available at 10.1186/s11689-022-09469-3.

## Background

Angelman syndrome (AS) is a neurodevelopmental disorder caused by different molecular mechanisms that eventually lead to the loss of function of the maternally inherited UBE3A gene in the 15q11-q13 chromosomal region [1]. The disease is characterized by ataxia, intellectual disability, speech impairment, seizures, hyperactivity, and happy demeanors [2]. Developmental outcomes of AS are associated with genotype including deletion and non-deletion type [3]. Epilepsy is present in more than 80% of affected individuals with AS. Most of the patients with AS have a first seizure at approximately 2 years old with a significant abnormal electroencephalographic background [4, 5]. Therefore, there is an increasing demand for a clear interpretation of the mechanism between seizures and AS.

Evidence from neural imaging studies has revealed the brain morphometry and functional changes in patients with AS. For example, previous studies have found a decrease in fractional anisotropy (FA) in the arcuate fasciculus [6, 7] and revealed severe atrophy of the striatum [8], which was associated with the language [9] and human intelligence development [10, 11]. Integrated quantitative MR imaging analysis showed poor functional and structural connectivity, as well as a reduction in brain volume in the children with AS compared to healthy controls (HC) [12]. Another study has indicated that the defects in subcortical structures and dysfunction of cortical networks were also related to the occurrence of epileptic seizures [13, 14]. In addition, both of the abnormal cortical thickness and folding have also been captured in epileptic seizure patients [15, 16]. A recent study suggested that the gyrification was related to human epilepsy and regulated by specific molecular mechanisms, such as the PIK3CA-related brain overgrowth [17]. In addition, the findings of the cortical morphological studies have also suggested an association between AS and UBE3A-related genetic disorders. For example, an accelerated gain in gray matter volume, which was caused by the attenuation of the cortical thickness (CTH) reduction was found in patients with autism [18]. Another recent study found abnormalities in cerebral cortical volume and thickness in patients with Prader-Willis syndromes (PWS) [19]. Moreover, the PWS, autism, and AS have all been demonstrated to be related to regional abnormalities on chromosome 15q11-13 [20]. These previous findings suggested that the brain structure of patients with AS was severely disrupted. Furthermore, previous studies have indicated that cortical gyrification was strongly associated with neurodevelopmental-related disorders, such as ASD [21–23] and Williams syndrome [24]. The cortical gyrification measured the regional cortical folding status and is usually estimated by using the local gyrification index (LGI) [25]. Since the gray matter volume could be seen as the product of CTH and cortical area [26], cortical folding also plays an important role in the relationship between CTH and volume. For example, cortical expansion and development result in cortical thinning and folding [27]. Abnormal cortical development may disrupt the relationship between these cortical morphometries. Therefore, a combination of different cortical morphological measurements would help to better understand the underlying mechanisms of AS brain abnormalities.

Based on previous brain morphological studies, our study aimed to discover the crucial structural alteration of AS and explore whether the combination of the volume, CTH, and LGI could provide an intuitive grasp of the difference in cortical or subcortical morphology. Furthermore, the interconnected structural changes may also help understand the key stages of brain development related to clinical characteristics.

## Methods

### Study participants

The study was designed as a cross-sectional study and was conducted under a research protocol. The participants were enrolled in the Department of Neurology in Children’s Hospital of Fudan University. The study was supported by the Chinese Angelman Syndrome Organization. The inclusion criteria for patients are as follows: (a) had been clinically and molecularly confirmed as AS, (b) had agreed and completed the magnetic resonance imaging (MRI) scan with sedation with oral chloral hydrate, and (c) quantified developmental delay according to Griffiths Mental Development Scales or Gesell Developmental Schedules. Genetic subtypes of AS include (a) maternal deletion of chromosome 15q11–13, (b) the maternally inherited UBE3A mutations, (c) UPD15pat, and (d) imprinting defect. Healthy age- and sex-matched control subjects were also enrolled in the same hospital, the control enrollment criteria include (a) agreed and completed the MRI scan, (b) had no neurological or psychiatric diagnoses and did not take any regular medications, and (c) had normal cognitive assessed using the Raven standard reasoning test score (IQ>80). This study was approved by the Children’s Hospital of Fudan University Institutional Review Board before the recruitment of subjects. Informed consent was obtained from the caregivers of the child participants.

Two experienced pediatric neurologists evaluated the seizure history and their electroencephalogram. Among these patients, those who had never experienced one seizure were included in the group of AS patients with non-seizure (AS-NSe). Patients who had experienced at least one seizure which include in the AS with the seizure (AS-Se) group.

### MRI acquisition and data processing

The participants underwent a whole-brain MRI using a 3-Tesla GE scanner. Structural images were acquired using a BRAVO sequence, and the imaging parameters were as follows: flip angle = 12, resolution = 1 × 1 × 1 mm^3^, echo time = 3.29 ms, repetition time=8.34 ms, inversion time = 450 ms, and 256 × 256 image matrix with 160 slices in the sagittal plane. Healthy children in the control group did not require sedation and could understand the instructions to continue completing the scan. All participants in the AS group were sedated by using 10% oral chloral hydrate (H03180450; Shanghai; 50m/kg, with the maximum amount at 1g), which was performed by trained anesthesiologists according to standard institutional protocol. MRI scans were scheduled to be performed at 8-10pm in the Department of Radiology in the hospital, and all datasets were assessed for quality and considered acceptable for analysis. After the acquisition, the unqualified data were excluded from the final analysis. No scans were repeated due to movements or other artifacts.

A total of 34 HC and 106 patients with AS were enrolled in our study. Visual inspection was performed before the analysis. Sixteen patients with AS were firstly excluded from our further analysis due to the incomplete data acquisition, and 8 of them were excluded due to head movements. Five HC were excluded from our study due to different acquisition parameters, and three of them were excluded due to data quality. In order to hold constant the genotypic background, only patients with maternal deletion of chromosome 15q11–13 were included. Finally, 73 patients and 26 HC were included in this study.

We firstly divided the data into two cohorts, one including AS patients and age-matched HC, and the other one including the AS patients with non-seizure and age-matched AS patients with seizures. For each cohort, an age-specific tissue template was created in the COM toolbox [28] by using the age-matched method. T1-weighted image processing was implemented in SPM12 (www.fil.ion.ucl.ac.uk) and the CAT12 toolbox in the MATLAB (www.mathworks.com). The T1-weighted images were first reoriented to the same orientation by aligning the AC-PC line. All of the images were corrected for bias field inhomogeneity and then segmented into gray matter, white matter, and cerebrospinal fluid by applying the study-specific tissue template. Segmented tissue maps were then spatially normalized to the MNI space by using the DARTEL algorithm [29]. Finally, the preprocessed gray matter data were smoothed with an 8-mm full-width half-maximum (FWHM) isotropic Gaussian kernel.

For surface-based morphometry (SBM), a pre-processing pipeline was adopted in the CAT 12 toolbox. A projection-based thickness estimation was used to measure the cortical thickness and reconstruct the central surface in one step [30]. The cortical thickness was evaluated by calculating the white matter distance based on tissue segmentation. This process also includes corrections for partial volume, sulcal blurring, and sulcal asymmetries. The gyrification index (GI) was extracted based on an absolute mean curvature approach [31]. The central cortical surfaces were created separately for both hemispheres. Finally, all surface measurements were resampled and smoothed using a Gaussian kernel of 15 mm (FWHM).

For volumetry analysis, the regions of interest (ROI) were selected from the results of the VBM and SBM analyses for further volumetric calculations. The bilateral inferior precuneus was selected for further analysis. In addition, we extracted the atlas-based ROIs defined by the WFU Pick Atlas [32] and the surface-based ROIs defined by DKT 40. We selected three sub-divisions of the caudate (head, body, and tail) and the precuneus for further analysis. The mean value of each morphological parameter was extracted from the selected ROIs for further volumetric analyses.

### Statistic analysis

To assess whether the morphological parameters differed between the groups, we adopted an independent two-sample *t* test between the HC and the AS groups and also between the AS-Se group and the AS-NSe groups. Age, gender, and total intracranial volume (TIV) were adjusted as covariates for each VBM or SBM comparison. Specifically, the TIV was adjusted as a proportional scaling for each image rather than as a covariate. To correct for multiple comparisons when using neuroimaging data, we also performed the SPM FWE peak correction, in which the clusters of *p* value < 0.05 and larger than 10 voxels or 10 vertices were considered significant. For the comparison between the AS-Se and the AS-NSe groups, an uncorrected threshold *p* value of < 0.005 was applied, and clusters less than 100 voxels or 100 vertices were excluded from each analysis. Cluster labeling was performed using SPM and the CAT12 toolbox. More specifically, the VBM results were labeled using the AAL atlas, and the SBM results were labeled using the DKT-40 atlas.

For volumetric and ROI analyses, we examined the effect of ROI morphological metrics while controlling for age, gender, and TIV. Before volumetric analysis, we performed the Winsorization technique to minimize the outliers. Multiple ordinary least squares (OLS) linear regression in R was used to fit the regression model:

*V* = 1 +β1*age +β2*gender +β3*TIV +β4*P (1), where *V* is the morphological metrics of ROIs, age, gender, and TIV are the covariates, and the *P* is the independent variable, such as the group variable and the regional morphometrics. For each regression model, a *p* value of <0.05 was considered significant. To avoid the multicollinearity between the predictors in the regression models. We calculated the variance inflation factors (VIFs) for age, TIV, and sex. The results demonstrated that all VIFs were well below the conservative threshold of 5 [33].

## Results

### Demographic characteristics and grouping

In this study, 73 patients with AS and 26 HC were included in the final analysis. The genetic mechanisms of these patients are also summarized in Table 1. Firstly, 32 patients with AS (age 6.75±1.38; gender 16:16; GQ score 29.40±9.97 ) and 26 age- and gender-matched HC (age 7.1±1.63; gender 13:13) were compared to investigate the cortical morphometry change of the AS patients, and all of these AS patients have reported experiencing a seizure. Secondly, 24 patients with seizure (age 2.69±0.5, gender 12:12, GQ score 30.48±5.55) and 17 patients with non-seizure (age 2.37±0.81, gender 7:10, GQ score 34.15±7.89) were compared to investigate the difference in brain morphometry between AS patients with seizure and non-seizure.

**Table 1:** Demographic of AS patients and healthy controls

	HC	AS	AS-Se	AS-NSe
Age (mean±SD)	7.1±1.63	6.75±1.38	2.69±0.5	2.37±0.81
*p* value	0.39		0.12	
Range	4.10–10.28	4.84–9.56	1.50–3.64	1.44–3.95
Gender				
Male	13	16	12	7
Female	13	16	12	10

*AS* Angelman patients, *HC* Health controls, *Se* Seizure, *NSe* Non-seizure

### VBM and SBM analysis for the AS-HC cohort

**Fig. 1:**
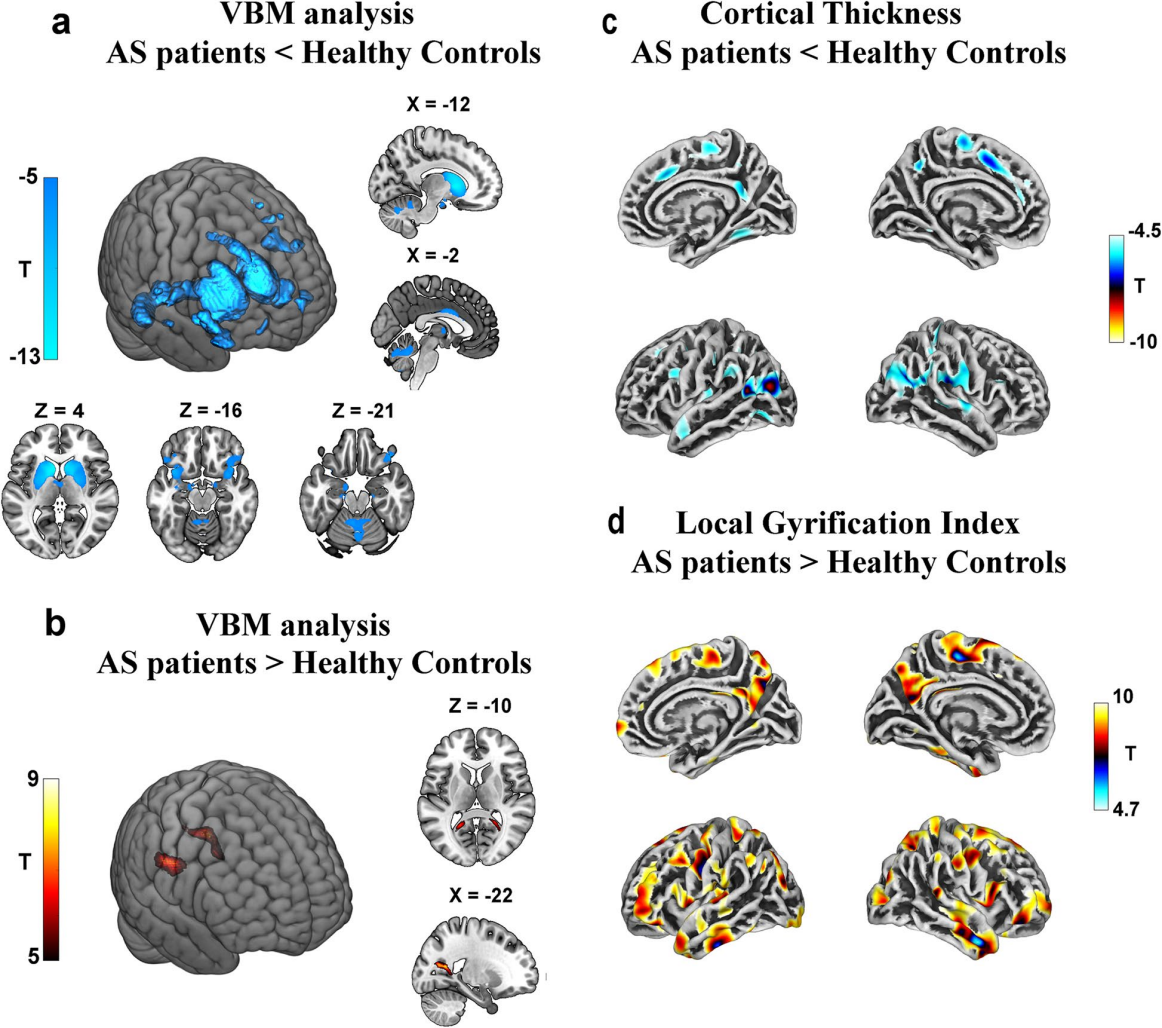
VBM and SBM analyses in AS-HC cohort showing by *T* statistic maps (reduced in blue and increased in red). VBM analysis showed significantly decreased gray matter volume in the subcortical nucleus and cerebellum (**a**) and significantly increased gray matter volume in the (**b**). The SBM analysis showed significantly reduced CTH (**c**) and significantly increased LGI across whole-brain in patients with AS when compared with HC (**d**). The results were thresholded at *p* < 0.05 in FWE peak correction, and clusters with less than 10 vertexes were eliminated. AS Angelman syndrome, HC health controls, Se seizure, NSe non-seizure, CTH cortical thickness, LGI local gyrification index

A significant reduction in gray matter volume in the subcortical areas was found in the AS group, including the caudate, the putamen, the pallidum, the amygdala, and the thalamus. The $\textrm{decrease}$ of gray matter volume was also found in the cortical and the cerebellum, such as the insula, the orbital frontal cortex, and the cerebellum dentates (FWE *p* < 0.05)(Fig. 1a). Moreover, the gray matter volume in patients with AS in the bilateral precuneus, which is located near the posterior horn of the lateral ventricle, was significantly increased (FWE *p* < 0.05) (Fig. 1b, Table S1).

The results of the group comparison revealed that the significant cluster with a largely decreased CTH of the AS group was located mainly in the bilateral inferior parietal, while other smaller clusters were located in the regions including the left superior frontal gyrus, left occipital, left insula, bilateral superior temporal gyrus, bilateral caudal middle frontal, right postcentral, right superior parietal, right supramarginal, and left precuneus (FWE *p* < 0.05) (Fig. 1c, Table S2).

Individuals with AS had a significantly increased in LGI in the whole brain as compared to HC. These clusters were distributed in areas including the anterior and the middle temporal cortex, the superior frontal cortex, the prefrontal cortex, the parietal cortex, the occipital cortex, and the precuneus. No regions with a significant decrease in the LGI were found in the AS group (Fig. 1d, Table S3).

### VBM and SBM analysis for AS seizure—non-seizure cohort

**Fig. 2:**
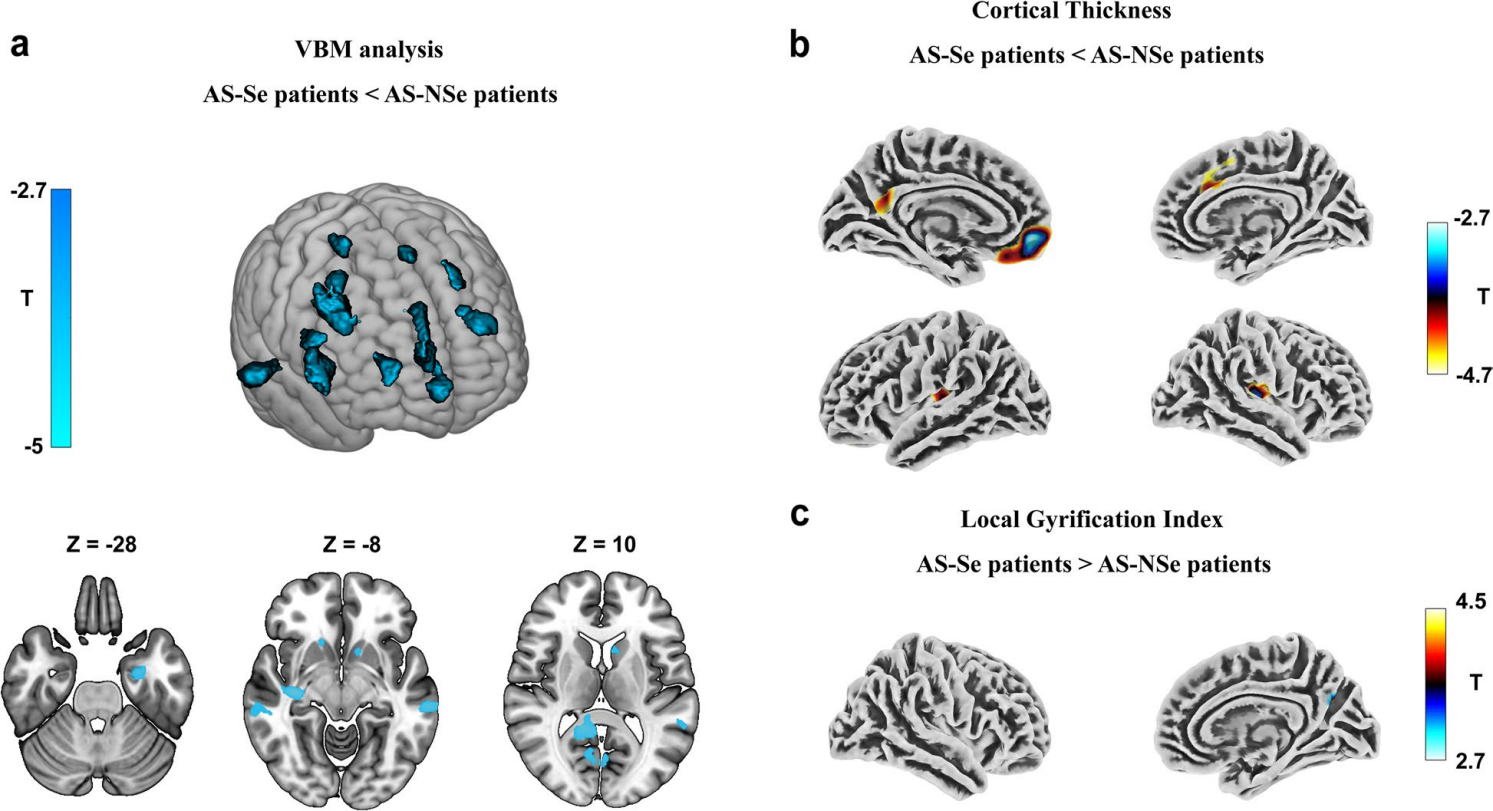
VBM and SBM analyses of seizure—non-seizure cohort showing by *T* statistic maps. (reduced in blue and increased in red). VBM analysis showed significantly reduced gray matter volume in bilateral caudate in AS-Se compared with AS-NSe groups (**a**). The results were thresholded at uncorrected *p* < 0.005 with clusters less than 100 voxels excluded. The SBM analysis showed significantly reduced CTH in the right superior frontal gyrus, left orbitofrontal gyrus, bilateral insula, and left precuneus (**b**) and significantly increased LGI in the right precuneus in the AS-Se group (**c**). The results were thresholded at uncorrected *p* < 0.005 and clusters’ size > 100 vertexes. AS Angelman syndrome, HC health controls, Se seizure, NSe non-seizure, CTH cortical thickness, LGI local gyrification index

The gray matter volume was reduced in the bilateral caudate, the insula, and the transverse temporal gyrus of the AS-Se group when compared to the AS-NSe group (Fig. 2a). The SBM-based comparison between the AS-Se and the AS-NSe revealed a smaller difference. The CTH in the right superior frontal gyrus, the left middle frontal gyrus, the left precuneus, and the bilateral insula were reduced in the AS-Se group (Fig. 2b). Furthermore, compared with the AS-NSe, we found that AS-Se had a mildly increased LGI in the right precuneus (Fig. 2c).

### Group differences in cortical thickness, GI, and density in the precuneus and caudate

**Fig. 3:**
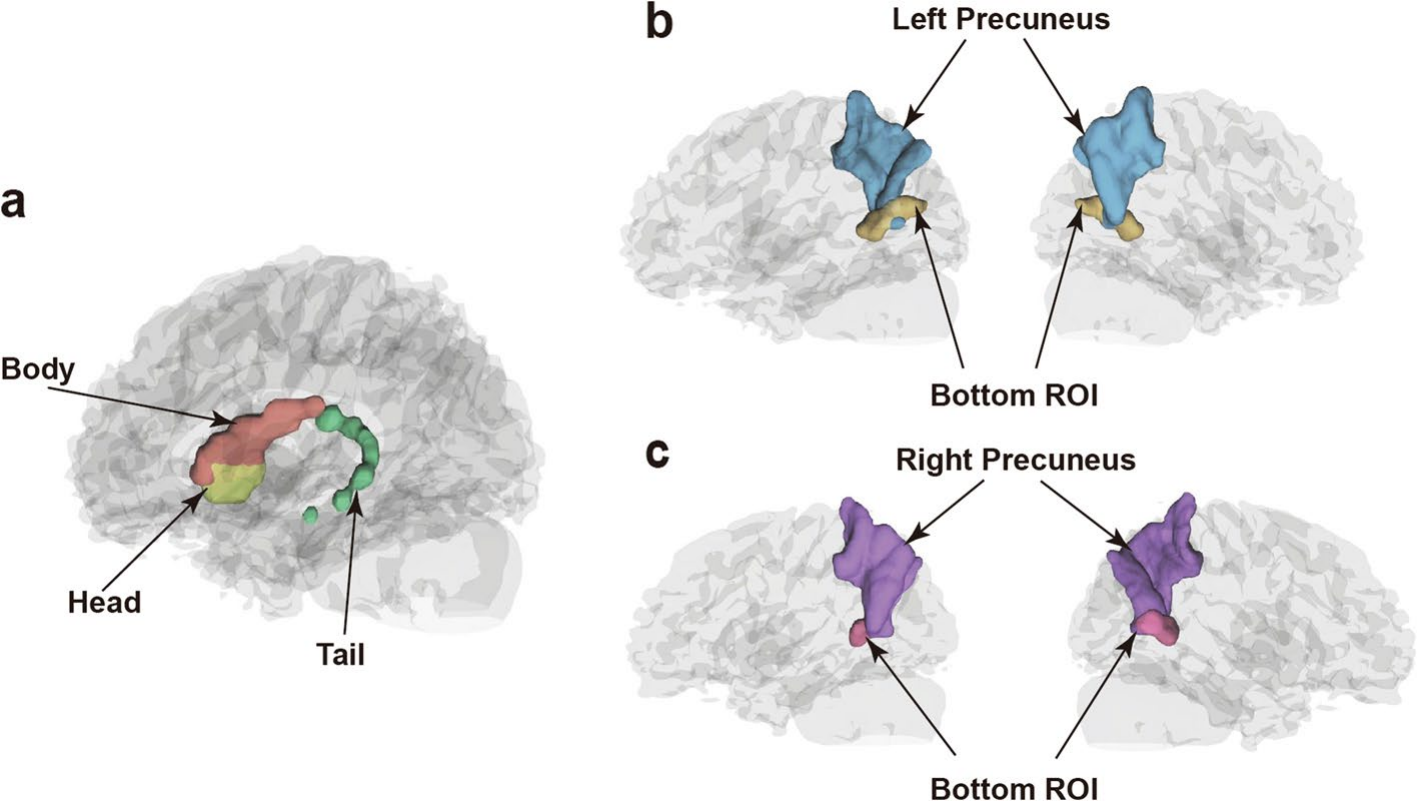
The ROI of volumetry analysis. The three subdivisions of the caudate nucleus, including the head, body, and tail (**a**). The left (**b**) and the right (**c**) ROI in the precuneus, including the precuneus and the inferior precuneus with an abnormal increase in volume in the AS group. ROI regions of interest

In addition, we further selected the caudate and precuneus as ROIs for the volumetric analysis, considering that a significant decrease in brain volume was centered on the caudate, and the co-alternation of morphometry was found in the precuneus. Specifically, the caudate was divided into three subdivisions, including the tail, head, and body by using WFU Pickatlas software [32]. The precuneus and the inferior precuneus with an abnormal increase in volume in the AS group were also included as ROIs (Fig. 3).

**Fig. 4:**
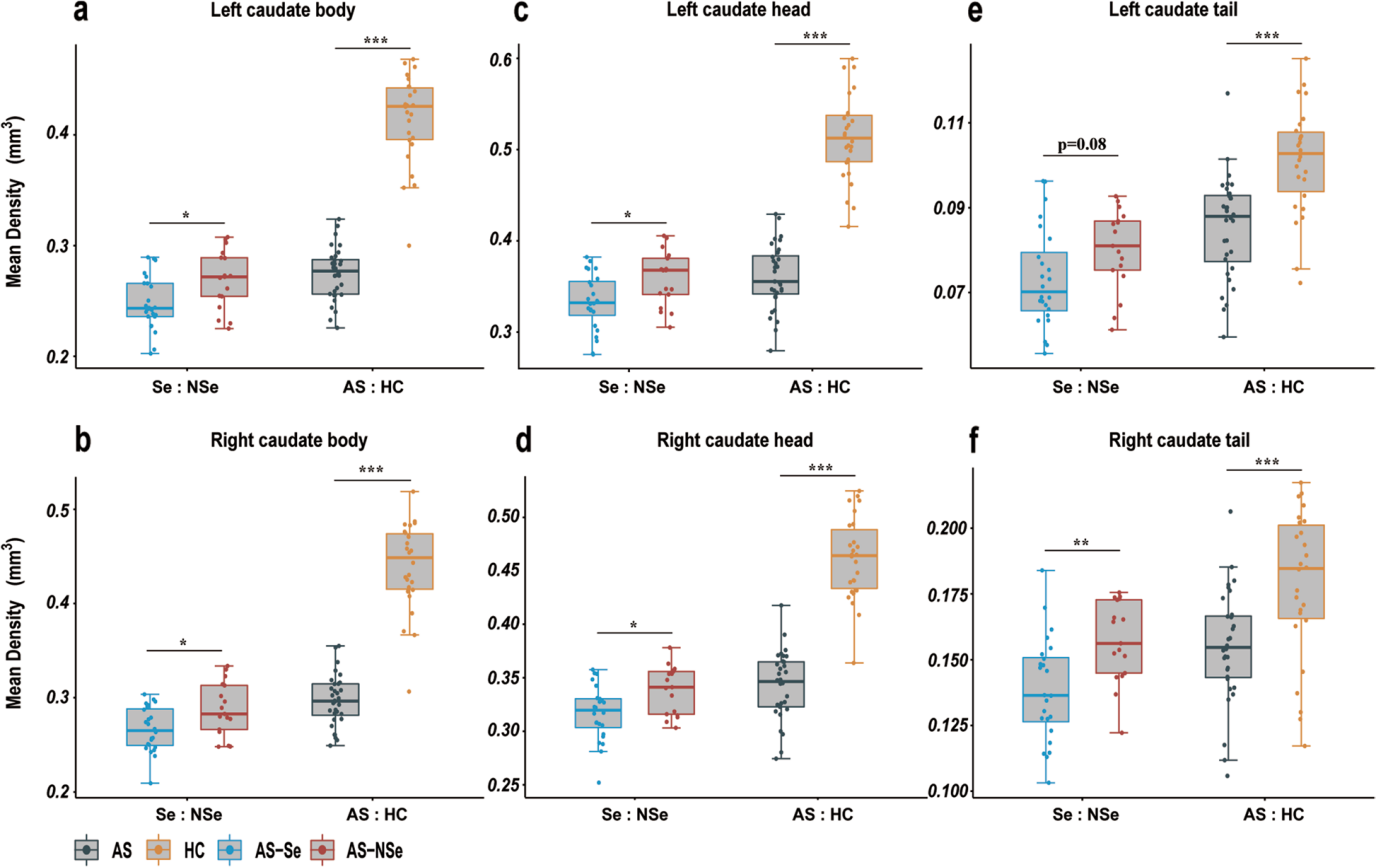
Quantitative analyses of caudate subdivisions. Boxplots of the mean densities in the three caudate subdivisions, including the bilateral caudate body (**a**, **b**), left and right caudate head (**c**, **d**), and left and right caudate tail (**e**, **f**). Age, gender, and TIV were included as covariates in the linear regression model, and each point represented a subject’s partial residual. ***p< 0.001, **p < 0.01, and *p<0.05. AS Angelman syndrome, HC health controls, Se seizure, and NSe non-seizure

Compared with the AS-NSe group, the AS-Se group exhibited a slightly lower density of the right caudate tail (*R*^2^=0.41, df=36, *p*=0.005, Cohen’s *f*^2^ group=0.24). In comparisons of the caudate head and body in the AS-Se group, we found that the mean density in the left and right caudate body was significantly reduced (left: *R*^2^=0.24, df=36, *p*=0.02, Cohen’s *f*^2^ group= 0.17; right: *R*^2^=0.27, df=36, *p*=0.02, Cohen’s *f*^2^ group= 0.17). The group difference in the subdivisions of the caudate head was also found to be significant (left: *R*^2^=0.25, df=36, *p*=0.02, Cohen’s *f*^2^ group= 0.17; right: *R*^2^=0.37, df=36, *p*=0.01, Cohen’s *f*^2^ group= 0.18) (Fig. 4).

**Fig. 5:**
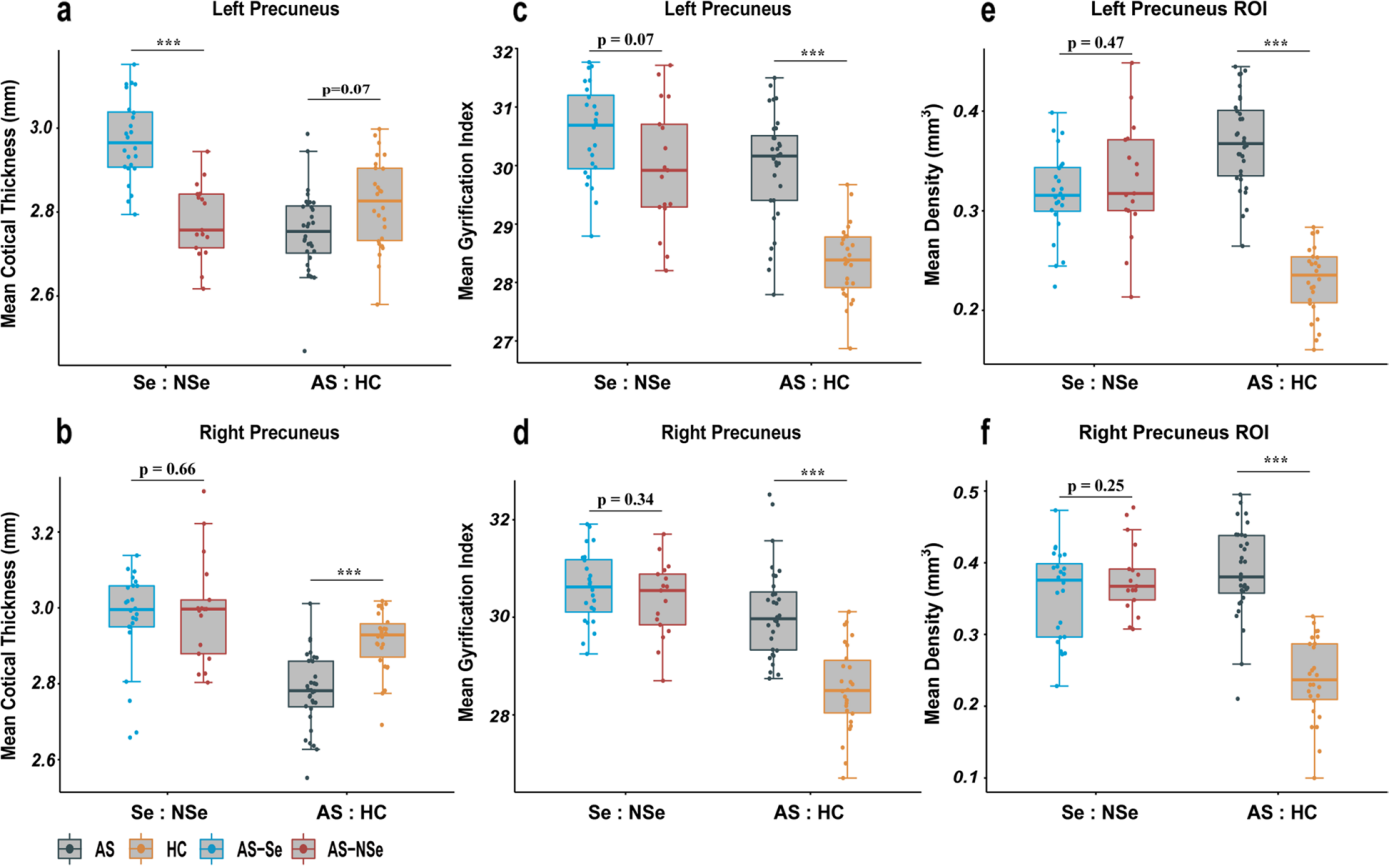
Quantitative analyses of precuneus morphometry metrics. Boxplots of the mean metrics in the three caudate subdivisions, including the left and right CTH of the precuneus (**a**, **b**), left and right LGI precuneus (**c**, **d**), and left and right (**e**, **f**). Age, gender, and TIV were included as covariates in the linear regression model, and each point represented a subject’s partial residual. ***p< 0.001, **p < 0.01, and *p<0.05. CTH cortical thickness, LGI local gyrification index, AS Angelman syndrome, HC health controls, Se seizure, NSe non-seizure

We also compared the differences in morphological metrics between the groups. Consistent with the SBM analysis, our results found that the AS group showed a significantly increased LGI in both the left and right precuneus compared to the HC group (left: *R*^2^=0.58, df=53, *p*-group<0.001, Cohen’s *f*^2^ group= 0.58; right: *R*^2^=0.65, df=53, p-group<0.001, Cohen’s *f*^2^ group= 0.43) and significantly reduced CTH in the right precuneus (*R*^2^=0.49, df=53, p-group<0.001, Cohen’s *f*^2^ group= 0.32). No significant differences were observed in the CTH in the left precuneus. The CTH of the left precuneus showed a significant difference between the AS-Se and the AS-NSe groups (*R*^2^=0.54, df=36, *p*<0.001, Cohen’s *f*^2^ group= 0.99). Our results also showed a significant difference in the left and right inferior precuneus between the AS and the HC groups (left: *R*^2^=0.65, df=53, *p*<0.001, Cohen’s *f*^2^ group= 1.62; right: *R*^2^=0.48, df=53, *p*<0.001, Cohen’s *f*^2^ group= 0.88) (Fig. 5).

## Discussion

In this study, we found that children with AS had thinner and more folded cortex than HC. Moreover, a reduction in brain volume in the subcortical nucleus has also been demonstrated in patients with AS, especially in patients with seizures. Remarkably, an increase in regional gray matter volume was observed in the precuneus in the AS group. To the best of our knowledge, this is the first study conducted on a Chinese population with AS.

The results of the VBM analysis indicated a significant decrease in gray matter volume in the subcortical nucleus of patients with AS, which is consistent with the previous study [8]. It has been demonstrated that the development of the subcortical nucleus volume was associated with individual factors [34], and the caudate was related to many diseases. For example, a recent study has suggested that the caudate was involved in restricted repetitive behaviors in autism [35]. An fMRI study indicated that the caudate and the putamen played an important role in language control [36]. Furthermore, the caudate volume has been found to be associated with cortical thickness and gyrification in previous studies of different diseases [37–39]. Interestingly, the decrease of caudate volume was more serious in patients with early seizures with a small-scale change in the cortical morphometry at the same time. Given that the relationship among caudate, cortex, and seizure has been identified in previous studies [40, 41], the caudate should be considered an important node for the alteration of the cortical structure in AS.

A large decrease in CTH was identified in the superior frontal cortex, temporal cortex, and supramarginal gyrus of the patients with AS. Specifically, the supramarginal gyrus located near the angular gyrus has been shown to have language-related functions [42]. Therefore, a decrease of thickness in these areas may directly damage language-related development in patients with AS. Furthermore, we also found variations in the precuneus CTH in the AS group. The precuneus was a deeply buried brain structure and has been found to be a human-specific, highly evolved region [43]. The precuneus has also been shown to be involved in several human brain networks and is responsible for the network communication [43]. Because the precuneus is an important node in the brain network [44], impairment of the structure in this area could directly or indirectly induce a variety of network disorders, such as seizures. Moreover, damage to the precuneus structure can induce several functional deficits, such as impaired autobiographical memory [45] and schizophrenia [46].

Other important findings in our study included an increase in LGI in the whole brain and an increase in gray matter volume in the inferior precuneus. To better adapt to the expansion of the human brain, the cortex continuously folds and gradually thins, thus increasing the surface area [47]. As a result, the regional gray matter volume accumulated throughout the process. However, we only observed gray matter accumulation in the precuneus. One reasonable explanation for this is that the location of the precuneus is associated with cortical expansion. The precuneus, located at the junction between the occipital cortex and the parietal cortex, can be divided into the sub-parietal sulcus, the parietal-occipital sulcus, and the cingulate sulcus [43]. Therefore, the gray matter can be easily accumulated in the corner in the case of a highly constrained space. Besides, a similar alternative of higher LGI has also been found in the Willams syndrome [24]. WS was a typical genetic mutation disorder characterized by intellectual disability, motor dysfunction, and myelination deficits [48]. Notably, the white matter impairment has also been found in AS-related studies [49, 50]. As the regional brain tissues were distributed by a kind of ratio between gray matter and white matter, it can be suggested that the regional increase in CTH may be associated with an alteration of the white matter structure. Prior rhetorical studies have shown that the white matter could play an important role in cortical folding, such as the fiber tension theory [51], and the complete absence of cortical fibers induces cortical folding as an effect of buckling instability [52]. A recent study using materials simulation has indicated that the gray matter was under compression, while the white matter was under radial tension beneath the gyri and circumferential tension beneath the sulci [27]. In summary, these theories and studies suggested that the formation of brain morphometry is strongly associated with the features of regional tissues.

Another reasonable interpretation of the gray matter overaccumulation in the precuneus was the specific genetic mutation. A recent study has determined the unique role of the big potassium channel (bK) in AS [53]. Due to the decrease in UBE3A expression level, the ubiquitination of the bK was interrupted, and thus, the frequency of hyperpolarization was also increased. As a result, the neuronal firing frequency was increased, suggesting an increased risk of epilepsy. It is also noteworthy that a recent study has indicated that the gyrification was related to sodium channel dysfunction [54]. The potassium and sodium flux were strongly associated with each other during the neuronal activity, and the higher level of gyrification in the precuneus may be associated with the potassium-related ion channel expression level in it.

### Limitations and future directions

It should be noted that the AS patients with seizure and non-seizure samples lacked HC, due to the difficulty in collecting data from healthy children under anesthesia. Besides, there was also lack of comparison with genetic subtypes of AS. Therefore, the data acquired in this study could not better interpret the brain morphometric change in early-stage of AS patients and the genetic subtypes of AS. Further research will collect more data from other imaging modalities, such as diffusion tensor imaging and functional imaging.

## Conclusion

These results revealed cortical and subcortical morphological alterations in patients with AS, including global volume reduction in the subcortical nucleus, the increased gray matter volume in the precuneus, and the whole-brain increase of LGI and reduction of CTH. The abnormal brain pattern was more serious in patients with seizures, suggesting that the occurrence of seizures may be related to abnormal brain changes.

## Supplementary Information

**Additional file 1.**

## Data Availability

The code for the draft is provided by the authors at https://github.com/HusBunia/RPlot. Keypoint data are available for reasonable request.

## Abbreviations

AS: Angelman syndrome
CTH: Cortical thickness
LGI: Local gyrification index
VBM: Voxel-based morphometry
SBM: Surface-based morphometry
ROI: Regions of interest
AS-Se: Angelman syndrome patients with seizure
AS-NSe: Angelman syndrome patients with non-seizure
PWS: Prader-Willis syndrome

## References

[1] Matsuura T, Sutcliffe JS, Fang P, Galjaard RJ, Jiang YH, Benton CS, Rommens JM, Beaudet AL (1997). De novo truncating mutations in E6-AP ubiquitin-protein ligase gene (UBE3A) in Angelman syndrome. Nat Genet.

[2] Margolis SS, Sell GL, Zbinden MA, Bird LM (2015). Angelman syndrome. Neurotherapeutics.

[3] Du X, Wang J, Li S, Ma Y, Wang T, Wu B, et al. An analysis of phenotype and genotype in a large cohort of Chinese children with Angelman syndrome. *Genes (Basel)*. 2022;13(8). 10.3390/genes13081447. 10.3390/genes13081447 PMC9408022 36011358

[4] Thibert RL, Conant KD, Braun EK, Bruno P, Said RR, Nespeca MP (2009). Thiele EA: Epilepsy in Angelman syndrome: a questionnaire-based assessment of the natural history and current treatment options. Epilepsia.

[5] Samanta D (2021). Epilepsy in Angelman syndrome: a scoping review. Brain Dev.

[6] Wilson BJ, Sundaram SK, Huq AH, Jeong JW, Halverson SR, Behen ME, Bui DQ, Chugani HT (2011). Abnormal language pathway in children with Angelman syndrome. Pediatr Neurol.

[7] Peters SU, Kaufmann WE, Bacino CA, Anderson AW, Adapa P, Chu Z, Yallampalli R, Traipe E, Hunter JV, Wilde EA (2011). Alterations in white matter pathways in Angelman syndrome. Dev Med Child Neurol.

[8] Aghakhanyan G, Bonanni P, Randazzo G, Nappi S, Tessarotto F, De Martin L, Frijia F, De Marchi D, De Masi F, Kuppers B (2016). From cortical and subcortical grey matter abnormalities to neurobehavioral phenotype of Angelman syndrome: a voxel-based morphometry study. PLoS One.

[9] Dick AS, Tremblay P (2012). Beyond the arcuate fasciculus: consensus and controversy in the connectional anatomy of language. Brain.

[10] Voelbel GT, Bates ME, Buckman JF, Pandina G, Hendren RL (2006). Caudate nucleus volume and cognitive performance: are they related in childhood psychopathology?. Biol Psychiatry.

[11] MacDonald PA, Ganjavi H, Collins DL, Evans AC, Karama S (2014). Investigating the relation between striatal volume and IQ. Brain Imaging Behav.

[12] Yoon HM, Jo Y, Shim WH, Lee JS, Ko TS, Koo JH, Yum MS (2020). Disrupted functional and structural connectivity in Angelman syndrome. AJNR Am J Neuroradiol.

[13] Norden AD, Blumenfeld H (2002). The role of subcortical structures in human epilepsy. Epilepsy & behavior : E&B.

[14] Kramer MA, Cash SS (2012). Epilepsy as a disorder of cortical network organization. Neuroscientist.

[15] Bernhardt BC, Chen Z, He Y, Evans AC, Bernasconi N (2011). Graph-theoretical analysis reveals disrupted small-world organization of cortical thickness correlation networks in temporal lobe epilepsy. Cereb Cortex.

[16] Ronan L, Murphy K, Delanty N, Doherty C, Maguire S, Scanlon C, Fitzsimons M (2007). Cerebral cortical gyrification: a preliminary investigation in temporal lobe epilepsy. Epilepsia.

[17] Roy A, Skibo J, Kalume F, Ni J, Rankin S, Lu Y, et al. Mouse models of human PIK3CA-related brain overgrowth have acutely treatable epilepsy. *Elife*. 2015;4. 10.7554/eLife.12703. 10.7554/eLife.12703 PMC4744197 26633882

[18] Smith E, Thurm A, Greenstein D, Farmer C, Swedo S, Giedd J, Raznahan A (2016). Cortical thickness change in autism during early childhood. Hum Brain Mapp.

[19] Manning KE, Tait R, Suckling J, Holland AJ (2018). Grey matter volume and cortical structure in Prader-Willi syndrome compared to typically developing young adults. Neuroimage Clin.

[20] Buiting K (2010). Prader-Willi syndrome and Angelman syndrome. Am J Med Gen Part C, Seminars Med Gen.

[21] Wallace GL, Robustelli B, Dankner N, Kenworthy L, Giedd JN, Martin A (2013). Increased gyrification, but comparable surface area in adolescents with autism spectrum disorders. Brain.

[22] Ecker C, Andrews D, Dell'Acqua F, Daly E, Murphy C, Catani M, Thiebaut de Schotten M, Baron-Cohen S, Lai MC, Lombardo MV (2016). Relationship between cortical gyrification, white matter connectivity, and autism spectrum disorder. Cereb Cortex.

[23] Kohli JS, Kinnear MK, Fong CH, Fishman I, Carper RA, Muller RA (2019). Local cortical gyrification is increased in children with autism spectrum disorders, but decreases rapidly in adolescents. Cereb Cortex.

[24] Gaser C, Luders E, Thompson PM, Lee AD, Dutton RA, Geaga JA, Hayashi KM, Bellugi U, Galaburda AM, Korenberg JR (2006). Increased local gyrification mapped in Williams syndrome. Neuroimage.

[25] Schaer M, Cuadra MB, Tamarit L, Lazeyras F, Eliez S (2008). Thiran JP: A surface-based approach to quantify local cortical gyrification. IEEE Trans Med Imaging.

[26] Gautam P, Anstey KJ, Wen W, Sachdev PS, Cherbuin N (2015). Cortical gyrification and its relationships with cortical volume, cortical thickness, and cognitive performance in healthy mid-life adults. Behav Brain Res.

[27] Tallinen T, Chung JY, Biggins JS, Mahadevan L (2014). Gyrification from constrained cortical expansion. Proc Natl Acad Sci U S A.

[28] Wilke M, Altaye M, Holland SK, Consortium CA (2017). CerebroMatic: a versatile toolbox for spline-based MRI template creation. Front Comput Neurosci.

[29] Ashburner J (2007). A fast diffeomorphic image registration algorithm. Neuroimage.

[30] Dahnke R, Yotter RA (2013). Gaser C: Cortical thickness and central surface estimation. Neuroimage.

[31] Luders E, Thompson PM, Narr KL, Toga AW, Jancke L, Gaser C (2006). A curvature-based approach to estimate local gyrification on the cortical surface. Neuroimage.

[32] Maldjian JA, Laurienti PJ, Kraft RA, Burdette JH (2003). An automated method for neuroanatomic and cytoarchitectonic atlas-based interrogation of fMRI data sets. Neuroimage.

[33] O’brien RM (2007). A caution regarding rules of thumb for variance inflation factors. Quality & Quantity.

[34] Potvin O, Mouiha A, Dieumegarde L, Duchesne S (2016). Normative data for subcortical regional volumes over the lifetime of the adult human brain. Neuroimage.

[35] Qiu T, Chang C, Li Y, Qian L, Xiao CY, Xiao T, Xiao X, Xiao YH, Chu KK, Lewis MH (2016). Two years changes in the development of caudate nucleus are involved in restricted repetitive behaviors in 2-5-year-old children with autism spectrum disorder. Dev Cogn Neurosci.

[36] Hervais-Adelman A, Moser-Mercer B, Michel CM, Golestani N (2015). fMRI of simultaneous interpretation reveals the neural basis of extreme language control. Cereb Cortex.

[37] Saleh A, Potter GG, McQuoid DR, Boyd B, Turner R, MacFall JR, Taylor WD (2017). Effects of early life stress on depression, cognitive performance and brain morphology. Psychol Med.

[38] Fortea J, Sala-Llonch R, Bartres-Faz D, Bosch B, Llado A, Bargallo N, Molinuevo JL (2010). Sanchez-Valle R: Increased cortical thickness and caudate volume precede atrophy in PSEN1 mutation carriers. J Alzheimers Dis.

[39] Zhang Y, Zhang J, Xu J, Wu X, Zhang Y, Feng H, Wang J, Jiang T (2014). Cortical gyrification reductions and subcortical atrophy in Parkinson's disease. Mov Disord.

[40] Vuong J, Devergnas A (2018). The role of the basal ganglia in the control of seizure. J Neural Transm (Vienna).

[41] Aupy J, Wendling F, Taylor K, Bulacio J, Gonzalez-Martinez J, Chauvel P (2019). Cortico-striatal synchronization in human focal seizures. Brain.

[42] Stoeckel C, Gough PM, Watkins KE, Devlin JT (2009). Supramarginal gyrus involvement in visual word recognition. Cortex.

[43] Cavanna AE, Trimble MR (2006). The precuneus: a review of its functional anatomy and behavioural correlates. Brain.

[44] Utevsky AV, Smith DV, Huettel SA (2014). Precuneus is a functional core of the default-mode network. J Neurosci.

[45] Ahmed S, Irish M, Loane C, Baker I, Husain M, Thompson S, Blanco-Duque C, Mackay C, Zamboni G, Foxe D (2018). Association between precuneus volume and autobiographical memory impairment in posterior cortical atrophy: beyond the visual syndrome. Neuroimage Clin.

[46] Gong X, Lu W, Kendrick KM, Pu W, Wang C, Jin L, Lu G, Liu Z, Liu H, Feng J (2014). A brain-wide association study of DISC1 genetic variants reveals a relationship with the structure and functional connectivity of the precuneus in schizophrenia. Hum Brain Mapp.

[47] Panizzon MS, Fennema-Notestine C, Eyler LT, Jernigan TL, Prom-Wormley E, Neale M, Jacobson K, Lyons MJ, Grant MD, Franz CE *et al*: Distinct genetic influences on cortical surface area and cortical thickness. *Cerebral cortex (New York, NY : 1991)* 2009, 19(11):2728-2735. 10.1093/cercor/bhp026 10.1093/cercor/bhp026 PMC2758684 19299253

[48] Nir A, Barak B. White matter alterations in Williams syndrome related to behavioral and motor impairments. *Glia*. 2020. 10.1002/glia.23868. 10.1002/glia.23868 32589817

[49] Judson MC, Burette AC, Thaxton CL, Pribisko AL, Shen MD, Rumple AM, Del Cid WA, Paniagua B, Styner M, Weinberg RJ (2017). Decreased axon caliber underlies loss of fiber tract integrity, disproportional reductions in white matter volume, and microcephaly in Angelman syndrome model mice. J Neurosci.

[50] Tiwari VN, Jeong JW, Wilson BJ, Behen ME, Chugani HT (2012). Sundaram SK: Relationship between aberrant brain connectivity and clinical features in Angelman syndrome: a new method using tract based spatial statistics of DTI color-coded orientation maps. Neuroimage.

[51] Van Essen DC (1997). A tension-based theory of morphogenesis and compact wiring in the central nervous system. Nature.

[52] Toro R, Burnod Y (2005). A morphogenetic model for the development of cortical convolutions. Cerebral cortex (New York, NY 1991).

[53] Sun AX, Yuan Q, Fukuda M, Yu W, Yan H, Lim GGY, Nai MH, D'Agostino GA, Tran HD, Itahana Y (2019). Potassium channel dysfunction in human neuronal models of Angelman syndrome. Science.

[54] Smith RS, Kenny CJ, Ganesh V, Jang A, Borges-Monroy R, Partlow JN, Hill RS, Shin T, Chen AY, Doan RN (2018). Sodium channel SCN3A (Na(V)1.3) regulation of human cerebral cortical folding and oral motor development. Neuron.

