# Supplementary material for: Cortical and subcortical morphological alteration in Angelman syndrome
Source: J Neurodev Disord. 2023 Feb 14;15:7. doi: 10.1186/s11689-022-09469-3 (PMC9930225; doi:10.1186/s11689-022-09469-3)
Supplement: Supplementary file 1 — Additional file 1. [file 11689_2022_9469_MOESM1_ESM.docx]

Table.S1 The group comparison results of the VBM analysis

|  | | **MNI coordinates** | | |  | | |
| --- | --- | --- | --- | --- | --- | --- | --- |
| Region | L/R | X | Y | Z | P(cluster) | T | Cluster size |
| ***HC > age-matched AS*** | | | | | | | |
| Caudate | L | -10 | 12 | 3 | <0.001 | 12.98 | 6494 |
| Putamen/pallidum | L |  |  |  |  |  |  |
| Insula | L | -36 | 12 | -46 | <0.001 | 8.19 |  |
| Thalamus | - | 0 | -6 | 4 | <0.001 | 5.45 |  |
| Caudate | R | 10 | 14 | 2 | <0.001 | 10.98 | 5467 |
| Puatmen/pallidum |  | 20 | 21 | -10 | <0.001 | 6.52 |  |
| Cerebellum | L/R | 22 | -64 | -30 | <0.001 | 7.92 | 3511 |
| Postcentral | L | -57 | -15 | 10 | <0.001 | 6.95 | 581 |
| Superior temporal gyrus | L |  |  |  |  |  |  |
| Rolandic Operculum | L |  |  |  |  |  |  |
| Parahippocampal gyrus | L | -15 | -18 | -24 | <0.001 | 6.72 | 321 |
| Hippocampal | L |  |  |  |  |  |  |
| Anterior Cingulate gyrus | L | -4 | -2 | 30 | 0.001 | 6.52 | 334 |
| Middle cingulate gyrus | L |  |  |  |  |  |  |
| Inferior Orbital frontal gyrus | L | -48 | 26 | -15 | <0.001 | 5.99 | 222 |
| Inferior frontal Operculum | L | -56 | 9 | 16 | 0.002 | 6.36 | 115 |
| Parahippocampal gyrus | R | 18 | -16 | -24 | 0.002 | 6.13 | 109 |
| Hippocampus | R | 15 | 3 | -14 | 0.003 | 5.94 | 89 |
| Poscentral | L | -56 | -21 | 27 | 0.004 | 5.75 | 76 |
| Cerebellum | R | 0 | -64 | -46 | 0.006 | 5.65 | 54 |
| Fusiform | L | -32 | 2 | -38 | 0.008 | 5.48 | 46 |
| Inferior temporal gyrus | L |  |  |  |  |  |  |
| Postcentral | R | 66 | -10 | 15 | 0.012 | 5.44 | 30 |
| Middle frontal gyrus | L | -26 | 3 | 56 | 0.013 | 5.39 | 29 |
| Cerebellum | L | -26 | -42 | -24 | 0.020 | 5.25 | 16 |
| Middle frontal gyrus | L | -30 | 16 | 50 | 0.022 | 5.73 | 14 |
| ***HC < age-matched AS*** | | | | | | | |
| Precuneus | L | -22 | -62 | 15 | <0.001 | 8.41 | 362 |
| Precuneus | R | 22 | -50 | 12 | <0.001 | 7.54 | 201 |
| ***AS-Se > AS-NSe*** | | | | | | | |
| Cerebellum | R | 9 | -76.5 | -34.5 | 0.30* | 3.41 | 159 |
| Parahippocampal gyrus | L | -27 | -4.5 | -30 | 0.18^*^ | 3.79 | 272 |
| Middle temporal gyrus | R | 64.5 | -33 | -15 | 0.04^*^ | 4.37 | 719 |
| Hippocampus | R | 37.5 | -12 | -13.5 | 0.03^*^ | 4.50 | 701 |
| Middle temporal gyrus | L | -63 | -30 | -6 | 0.15^*^ | 3.81 | 312 |
| Caudate | L | -13.5 | 6 | 1.5 | 0.03^*^ | 4.47 | 755 |
| Caudate | R | 12 | 9 | -1.5 | 0.19^*^ | 3.65 | 258 |
| Parahippocampal gyrus | R | 33 | -37.5 | -3 | 0.23^*^ | 3.97 | 219 |
| Posterior cingulate gyrus | R | 9 | -49.5 | 6 | 0.01^*^ | 4.11 | 1097 |
| Lingual gyrus | L | -4.5 | -72 | 15 | 0.29^*^ | 3.76 | 165 |
| Superior temporal gyrus | L | -57 | -48 | 18 | 0.31^*^ | 3.12 | 152 |
| Cuneus | L | -12 | -87 | 24 | 0.28^*^ | 3.74 | 175 |
| Superior frontal gyrus | L | -6 | 3 | 60 | 0.013^*^ | 3.29 | 113 |

Abbreviations: AS, angelman syndromes; HC, healthy controls; equiv k, cluster size of volume. *, uncorrected cluster level p value.

Table.S2 The group comparison results of the cortical thickness

|  | | **MNI coordinates** | | |  | | |
| --- | --- | --- | --- | --- | --- | --- | --- |
| Region | L/R | X | Y | Z | P(cluster) | T | Cluster size |
| ***HC < age-matched AS*** | | | | | | | |
| Inferior parietral gyrus | L | -21 | -65 | 36 | <0.001 | 9.76 | 1200 |
| Superior parietal gyrus | L |  |  |  |  |  |  |
| Inferior parietal gyrus | R | 45 | -61 | 21 | <0.001 | 7.25 | 1037 |
| Superior parietal gyrus | R |  |  |  |  |  |  |
| Supramarginal gyrus | R | 50 | -26 | 20 | <0.001 | 7.12 | 603 |
| Postcentral | L |  |  |  |  |  |  |
| Superior frontal gyrus | R | -7 | 9 | 47 | <0.001 | 6.72 | 290 |
| Superior temporal gyrus | L | -38 | -22 | -4 | <0.001 | 6.61 | 330 |
| Insula | L |  |  |  |  |  |  |
| Superior parietal gyrus | R | 23 | -36 | 64 | <0.001 | 6.31 | 328 |
| Postcentral | R |  |  |  |  |  |  |
| Superior frontal gyrus | L | -5 | -14 | 63 | <0.001 | 6.19 | 134 |
| Paracentral | L |  |  |  |  |  |  |
| Superior temporal gyrus | R | 64 | -11 | 2 | <0.001 | 5.96 | 163 |
| Caudal medial frontal gyrus | L | -28 | -1 | 49 | <0.001 | 5.96 | 182 |
| Precentral | L |  |  |  |  |  |  |
| Superior frontal gyrus | R | 10 | 21 | 38 | <0.001 | 5.95 | 139 |
| Isthmus cingulate gyrus | R | 3 | -50 | 25 | <0.001 | 5.94 | 92 |
| Precuneus | R |  |  |  |  |  |  |
| Superior temporal gyrus | R | 56 | -30 | 0 | <0.001 | 5.88 | 118 |
| Bankssts | R |  |  |  |  |  |  |
| Paracentral | R | 6 | -21 | 61 | <0.001 | 5.65 | 147 |
| Superior frontal gyrus | R |  |  |  |  |  |  |
| Parsopercularis | R | 47 | 21 | 20 | <0.001 | 5.64 | 92 |
| Inferior parietal gyrus | R | 42 | -54 | 42 | <0.001 | 5.55 | 156 |
| Supramarginal gyrus | L | -54 | -38 | 29 | <0.001 | 5.43 | 150 |
| Lateral occipital cortex | L | -46 | -71 | -6 | <0.001 | 5.54 | 137 |
| Inferior temporal gyrus | L |  |  |  |  |  |  |
| Parsopercularis | L | -34 | -12 | 29 | <0.001 | 5.44 | 153 |
| Caudal medial frontal gyrus | L | -44 | 4 | 20 | <0.001 | 4.75 |  |
| Precuneus | L | -9 | -56 | 43 | 0.001 | 6.07 | 86 |
| Inferior parietal gyrus | L | -34 | -69 | 47 | <0.001 | 5.38 | 197 |
| Supramargninal gyrus | L |  |  |  |  |  |  |
| Postcentral | L | -50 | -26 | 42 | 0.003 | 5.22 | 55 |
| Superior frontal gyrus | L | -22 | 25 | 46 | 0.005 | 5.14 | 46 |
| Insula | R | 37 | -22 | -1 | 0.008 | 5.13 | 36 |
| Superior temporal gyrus | R |  |  |  |  |  |  |
| Fusiform | L | -33 | -47 | -10 | 0.017 | 4.96 | 21 |
| Supramarginal gyrus | L | -54 | -19 | 17 | 0.023 | 4.89 | 15 |
| Postcentral | L |  |  |  |  |  |  |
| Superior frontal gyrus | L | -7 | 46 | 29 | 0.019 | 4.89 | 19 |
| Caudal middle frontal gyrus | L | -31 | 19 | 45 | 0.009 | 4.87 | 33 |
| Precuneus | R | 6 | -48 | 45 | 0.014 | 4.84 | 25 |
| Superior parietal gyrus | L | -36 | -49 | 49 | 0.012 | 4.78 | 28 |
| Precuneus | R | 10 | -56 | -12 | 0.026 | 4.72 | 13 |
| Postcentral | L | -43 | -6 | 13 | 0.027 | 4.72 | 12 |
| Precentral | L |  |  |  |  |  |  |
| Superior parietal gyrus | L | -22 | -46 | 60 | 0.029 | 4.68 | 11 |
| ***AS-NSe > AS-Se*** | | | | | | | |
| Insula | L | 41 | -16 | -5 | 0.006^*^ | 3.70 | 231 |
| Precuneus | L | -7 | -53 | 23 | 0.126^*^ | 3.60 | 118 |
| Insula | R | 35 | -24 | 6 | <0.001^*^ | 4.56 | 608 |
| Superior frontal gyrus | R | 9 | 19 | 33 | 0.021^*^ | 3.46 | 220 |
| Middle orbital frontal gyrus | L | -6 | 51 | -8 | 0.008^*^ | 4.04 | 278 |

Abbreviations: AS, angelman syndromes; HC, healthy controls; *, uncorrected p value. AS-Se, the angelman patients with seizure;

AS-NSe, the angelman patients without seizure.

Table.S3 The group comparison results of the local gyrification index

|  | **MNI coordinates** | | | |  | | |
| --- | --- | --- | --- | --- | --- | --- | --- |
| Region | L/R | X | Y | Z | P(cluster) | T | Cluster size |
| ***HC > age-matched AS*** | | | | | | | |
| Middle temporal gyrus | R | 58 | 0 | -30 | <0.001 | 9.35 | 1059 |
| Superior temporal gyrus | R |  |  |  |  |  |  |
| Superior frontal gyrus | L | -5 | -20 | 55 | <0.001 | 9.07 | 609 |
| Paracentral | L |  |  |  |  |  |  |
| Superior temporal gyrus | L | -47 | -27 | 3 | <0.001 | 8.63 | 950 |
| Rostral medial frontal gyrus | L | -37 | 35 | -12 | <0.001 | 8.41 | 1382 |
| Caudal medial frontal gyrus | L |  |  |  |  |  |  |
| Inferior temporal gyrus | L | -56 | -23 | -33 | <0.001 | 8.34 | 301 |
| Middle temporal gyrus | L |  |  |  |  |  |  |
| Precentral | L | -42 | -13 | 33 | <0.001 | 8.21 | 1122 |
| Postcentral | L | -43 | -9 | 59 | <0.001 | 6.16 |  |
| Inferior parietal gyrus | L | -32 | -72 | 46 | <0.001 | 7.91 | 284 |
| Precuneus | R | 7 | -69 | 34 | <0.001 | 7.79 | 1169 |
| Parsobitalis | R | 48 | 38 | -14 | <0.001 | 7.67 | 260 |
| Rostral middle frontal gyrus | R |  |  |  |  |  |  |
| Precuneus | L | -3 | -62 | 20 | <0.001 | 7.60 | 523 |
| Parsopercuaris | R | 54 | 13 | 15 | <0.001 | 7.41 | 379 |
| Precentral | R |  |  |  |  |  |  |
| Precentral | L | -56 | 5 | 1 | <0.001 | 7.18 | 365 |
| Parsopercularis | L |  |  |  |  |  |  |
| Inferior parietal gyrus | R | 38 | -84 | 25 | <0.001 | 7.12 | 362 |
| Lateral occipital gyrus | R | 28 | -86 | 5 | <0.001 | 6.49 |  |
| Precentral | R | 40 | -13 | 40 | <0.001 | 7.07 | 582 |
| Postcentral | R | 53 | -19 | 36 | <0.001 | 6.53 |  |
| Superior frontal gyrus | R | 6 | -21 | 61 | <0.001 | 6.82 | 883 |
| Paracentral | R |  |  |  |  |  |  |
| Superior parietal gyrus | L | -24 | -48 | 59 | <0.001 | 6.82 | 308 |
| Rostral middle frontal gyrus | R | 10 | 67 | 0 | <0.001 | 6.82 | 196 |
| Precentral | R | 29 | -26 | 49 | <0.001 | 6.41 | 319 |
| Lateral occipital gyrus | L | -35 | -90 | -15 | <0.001 | 6.09 | 152 |
| Supramarginal gyrus | L | -43 | -52 | 40 | <0.001 | 5.98 | 159 |
| Superior parietal gyrus | R | 18 | -39 | 66 | <0.001 | 6.20 | 147 |
| Postcentral | R |  |  |  |  |  |  |
| Caudal middle frontal gyrus | R | 40 | 14 | 54 | <0.001 | 5.66 | 125 |
| Bankssts | R | 63 | -39 | 12 | <0.001 | 7.36 | 118 |
| Parahippocampal gyrus | R | 28 | -20 | 31 | <0.001 | 5.63 | 96 |
| Superior temporal gyrus | L | -51 | -8 | -17 | <0.001 | 5.67 | 82 |
| Inferior parietal cortex | L | -41 | -65 | 34 | <0.001 | 6.16 | 77 |
| Inferior temporal gyrus | L | -54 | -54 | 21 | <0.001 | 6.02 | 70 |
| Fusiform | L | -29 | -37 | -24 | <0.001 | 6.03 | 65 |
| Isthmus cingulate cortex | L | -4 | -37 | 25 | <0.001 | 6.35 | 63 |
| Posterior cingulate gyrus | L |  |  |  |  |  |  |
| Lateral orbital frontal gyrus | R | 13 | 24 | -23 | <0.001 | 6.14 | 79 |
| Lingual | R | -29 | -37 | -24 | <0.001 | 6.03 | 65 |
| Supramarginal gyrus | R | 58 | -37 | 41 | 0.001 | 5.55 | 45 |
| Postcentral | L | -43 | -23 | 54 | 0.001 | 5.23 | 38 |
| Superior parietal gyrus | L | -12 | -57 | 58 | 0.002 | 5.10 | 35 |
| Rostral middle frontal gyrus | R | 46 | 22 | 30 | 0.002 | 5.08 | 34 |
| Parstriangularis | L | -39 | 25 | 6 | 0.003 | 4.94 | 31 |
| Fusiorm | R | 34 | -26 | 11 | 0.010 | 4.94 | 17 |
| Superior frontal | R | 11 | 44 | 18 | 0.010 | 5.22 | 17 |
| Insula | R | 43 | -61 | -11 | 0.010 | 5.04 | 17 |
| Supramarginal gyrus | L | -54 | -53 | 24 | 0.018 | 4.9 | 11 |
| ***AS-Se > AS-NSe*** | | | | | | | |
| Precuneus | R | 10 | -66 | 27 | 0.027^*^ | 4.49 | 111 |

Abbreviations: AS, angelman syndromes; HC, healthy controls; AS-Se, the angelman patients with seizure;

AS-NSe, the angelman patients without seizure.
